# Medical School, Cardiovascular Surgery, and Education: how we do it in Brazil’s scenario?

**DOI:** 10.21470/1678-9741-2020-0365

**Published:** 2021

**Authors:** Tiago Santos Ribeiro, Renato Max Faria, Magaly Arrais dos Santos

**Affiliations:** 1 Onofre Lopes’s University Hospital, Universidade Federal do Rio Grande do Norte, Natal, Rio Grande do Norte, Brazil.; 2 Dante Pazzanese’s Institute of Cardiology, São Paulo, São Paulo, Brazil.

**Keywords:** Medical School, Students, Medical, Cardiac Surgery, Internship and Residency, Specialties, Surgical, Medicine

## Abstract

Choosing a surgical specialty can be a hard decision for a medical student. Several studies present data showing that most medical students fear the surgical field and end up switching to another specialty. For cardiovascular surgery, the scenario is very similar. In the last decades, the interest in cardiovascular surgery has been decreasing worldwide and the cardiothoracic surgical societies across the globe have been trying to understand the factors that push away medical students and general surgical residents from the specialty. In this regard, our work aims to focus on describe the access of students to cardiovascular surgery, especially during medical school, as well as to provide a brief report of our current data regarding the specialty.

**Table:** 

Abbreviations, acronyms & symbols	
BDALCS	= Brazilian Department of Academic Leagues in Cardiovascular Surgery
BJCVS	= Brazilian Journal of Cardiovascular Surgery
BSCVS	= Brazilian Society of Cardiovascular Surgery

## INTRODUCTION

The first residency program was established by Sir William Osler and Dr. William Halsted at the Johns Hopkins University School of Medicine. The so-called Halsted’s method involved training residents in surgical skills, patient care, and the evolution of the residents’ responsibility each year^[^^1^^]^. In the early 19s, John Crawford Crosby, an American lawyer and politician, said that “mentoring is a brain to pick, an ear to listen, and a push in the right direction”. From that perspective, it is well known that choosing a surgical specialty can be challenging for medical students who did not get the chance to actually live the reality of the career during medical school. So here it comes the role of a mentor: to guide highly motivated students through the hard journey that involves becoming a cardiovascular surgeon, propitiating an environment of knowledge, self-trust and confidence, allowing the mentee to grow both as a person and a future doctor.

Medical students’ will to pursue a career in cardiothoracic surgery is decreasing in the last decades. In fact, from a United Kingdom database, only 14% of the last year medical students considered a career in cardiothoracic surgery. Moreover, 75% of the students said that their exposure to cardiac surgery during medical school was poorly done and 74% claimed zero-time exposure to the field during college. Despite that, the factor that attracted medical students was the capability of improving the patients’ statuses, of saving lives, and the main negative factor was the highly competitive scenario, as well as other factors like the complexity of the area, the broad field, long hours away from home, training centers restricted to big cities and/or tertiary hospitals, stressful life (*i.e*., dealing with death constantly), and the senior surgeon’s behavior - in a demanding environment^[^^2^^]^.

In Brazil, the scenario is similar. The medical students’ interest in following a cardiovascular surgery pathway is low. The core curriculum exposure to cardiothoracic surgery in some universities is minimum to none, and the core clerkships doesn’t provide the adequate picture of the specialty. In addition, it is difficult for the student to practice cardiovascular science inside the operation field because of the complexity of the procedures.

In the last decade, the specialty suffered with low rates of filled positions in training centers. Traditionally, cardiac surgery in Brazil had two pathways: the first one included two years of general surgery training plus four years of cardiovascular surgery, accredited by our Ministry of Education, and the second was a four-year training after medical school at some institutions all over the country, supervised by the Brazilian Society of Cardiovascular Surgery (BSCVS). The main difference between them is the need for passing a cardiovascular surgery board exam in the second option to become a cardiovascular surgeon accredited by BSCVS.

In 2018, after years of debate, both BSCVS and the Ministry of Education created a five-year program of medical residency without the need for two years of general surgery, with the possibility of acquiring knowledge in the various areas of clinical and interventional cardiology, as well as in cardiac and endovascular surgery^[^^3^^]^. It is important to notice that unlike most countries, like the United States of America, the cardiovascular surgery residency program does not englobe training in the thoracic track, which is a different acknowledged specialty in our country.

The implementation of the five-year unified training increased the interest in cardiovascular surgery. For example, data from the Universidade de São Paulo, one of the most important training centers in Brazil, which offers six training positions each year for postgraduate year one, showed the increase from 3,6 candidates per position to 13,4 candidates per position in 2019, after the curriculum transition in 2018^[^^4^^]^. 

It is also worth mention that in Brazil, according to the last Federal Council of Medicine’s census, published in 2018, there were 2,271 board-certified cardiovascular surgeons, with only 10,4% of these being women, which is a disquieting number for the proportions of a country like Brazil^[^^5^^]^. Women are not encouraged to surgical specialties, since their entrance in Medical School, especially in cardiovascular surgery, which is a fact directly linked to a thought that female surgeons wouldn’t be capable of managing both professional and personal lives.

The smaller the number of students interested in cardiovascular surgery, the smaller the number of cardiovascular surgeons will be. In this context, creating strategies to attract students to the cardiovascular surgery pathway is a *sine qua non* condition to maintain the specialty alive. 

**Fig. 1:**
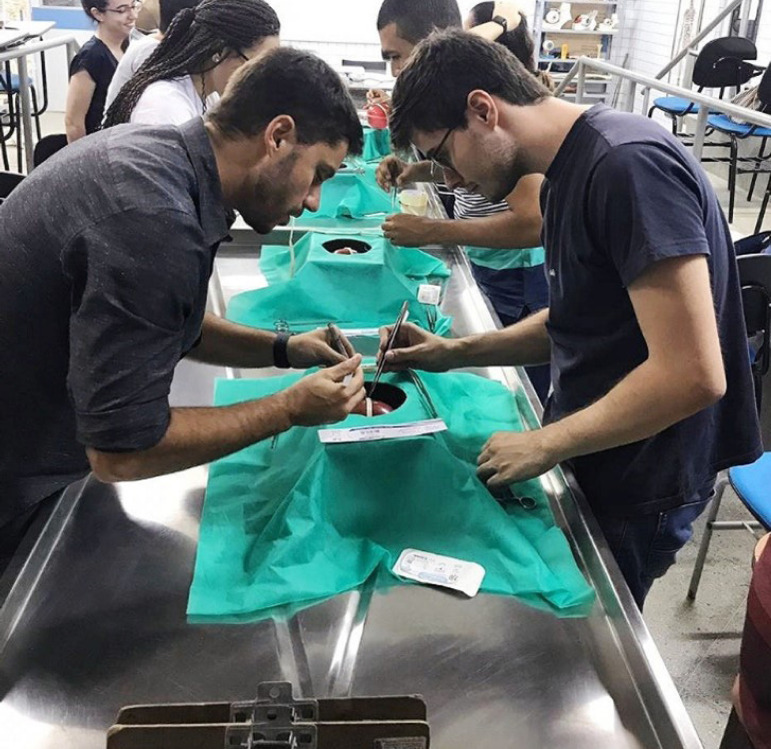
Cardiovascular surgeon, Dr. Renato Max, MD., teaching medical students how to perform a coronary vascular anastomosis using a lowcost material in Mossoró, a countryside city in Rio Grande do Norte, Brazil’s Northeast Region. Image courtesy from the authors.

Historically, Brazilian medical schools have created the so-called Academic Leagues (*i.e*., interest groups), which are academic institutions guided by medical students with certain affection for an field of Medicine and supervised by one specialist doctor^[^^6^^]^. In terms of cardiovascular surgery, Brazil counts with an academic organ linked to BSCVS, the Brazilian Department of Academic Leagues in Cardiovascular Surgery (BDALCS), an institution that is coordinated by medical students throughout the country, with 67 leagues registered at the moment of this publication. BDALCS is also extremely involved with BSCVS, promoting events dedicated to continued education, like the Itinerant Experimental Cardiovascular Surgery Training, in which the Academic Leagues members of the Department are oriented by their surgical mentors in basic procedures, such as vascular anastomosis, the basis of coronary artery bypass grafting, aortic surgery, and valvular procedures, with low-cost materials, as evidence shows that in-training laboratory experimental experience may increase medical students’ interest in the cardiothoracic field (Figure 1)^[^^7^^]^.

**Fig. 2:**
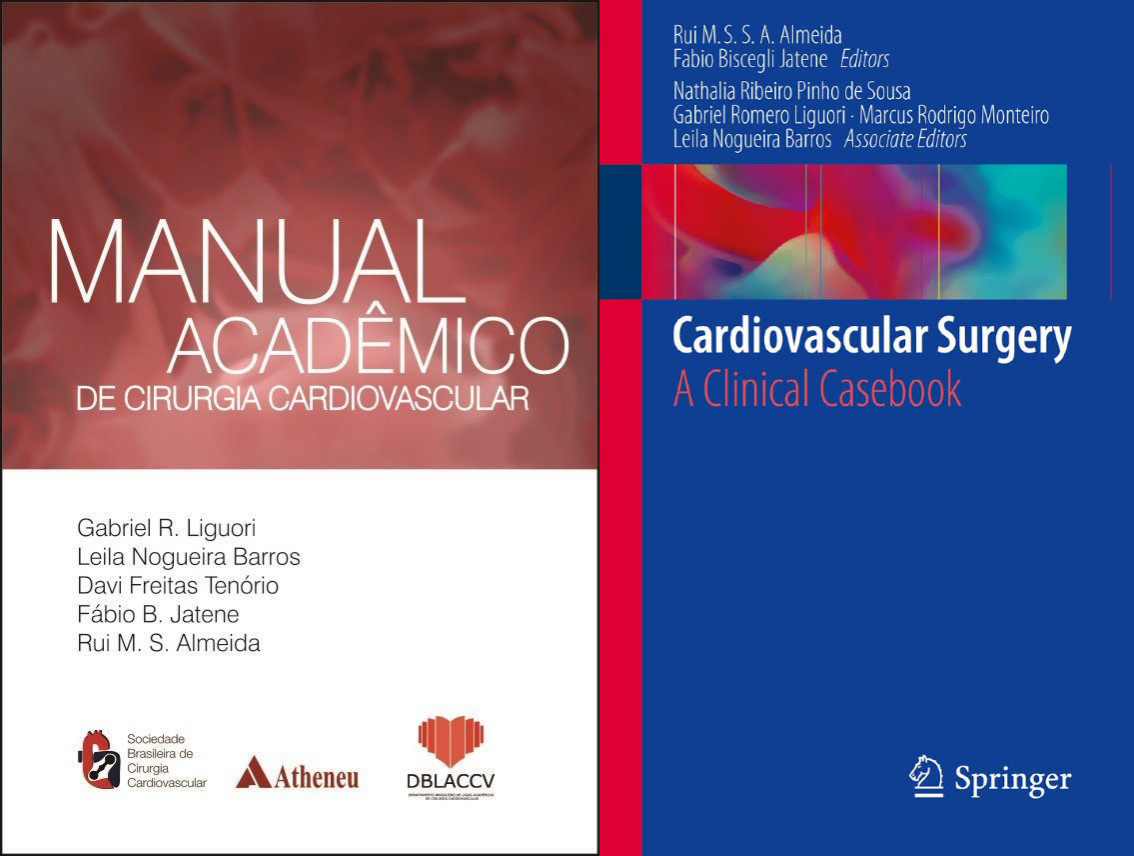
Books published by the Brazilian Department of Academic Leagues in Cardiovascular Surgery, in association with the Brazilian Society of Cardiovascular Surgery. The main authors are medical students supervised by graduated surgeons. Image courtesy from BDALCS.

Other opportunities given by the BDALCS include the creation of a data bank for future publications involving medical education and the already published two books, “Cardiovascular Surgery: A Clinical Casebook" and “Manual Acadêmico de Cirurgia Cardiovascular” (Figure 2).

Moreover, the Brazilian Journal of Cardiovascular Surgery (BJCVS), the official journal of the BSCVS, supports an online blog that publishes on a weekly basis frequency about a wide variety of themes related to cardiovascular surgery. Most of the editorial team are Junior Editors, a selected group of medical students with a career interest in cardiovascular surgery and research, under supervision of an editorial assistant and two editorial fellows that are surgical residents with research expertise, as well as other representants of BSCVS. Furthermore, the Editorial team of BJCVS blog is also responsible for keeping the BSCVS media updated, promoting surgical education and ensuring the quality of information delivered in all of our social media.

In addition, the Brazilian Congress of Cardiovascular Surgery gives to the students the chance of organizing the Academic Congress of Cardiovascular Surgery, in which students from all over the country are exposed to lectures given by specialists about career, leadership, and mentorship, and they are still able to perform oral presentations of papers selected by the BSCVS commission, with included awards to the best performances. This year, in the current situation of coronavirus pandemic and in respect to the students with approved oral papers, the Academic Congress was performed online. Moreover, a female cardiovascular surgeons meeting (the Encontro das Mulheres Cirurgiãs) happens each year in one of the Congress nights. The event, hosted by female cardiovascular surgeons in a leading position of their careers, is devoted to all women participant in the Brazilian Congress of Cardiovascular Surgery, mainly the female medical students and surgical residents, where they have the chance to talk about the many difficulty that women still finds in the residency and to develop the opportunity of having a mentorship and expanding career interests, demystifying the pathways to be a female surgeon and meeting real life examples of successful women in cardiovascular surgery.

**Fig. 3:**
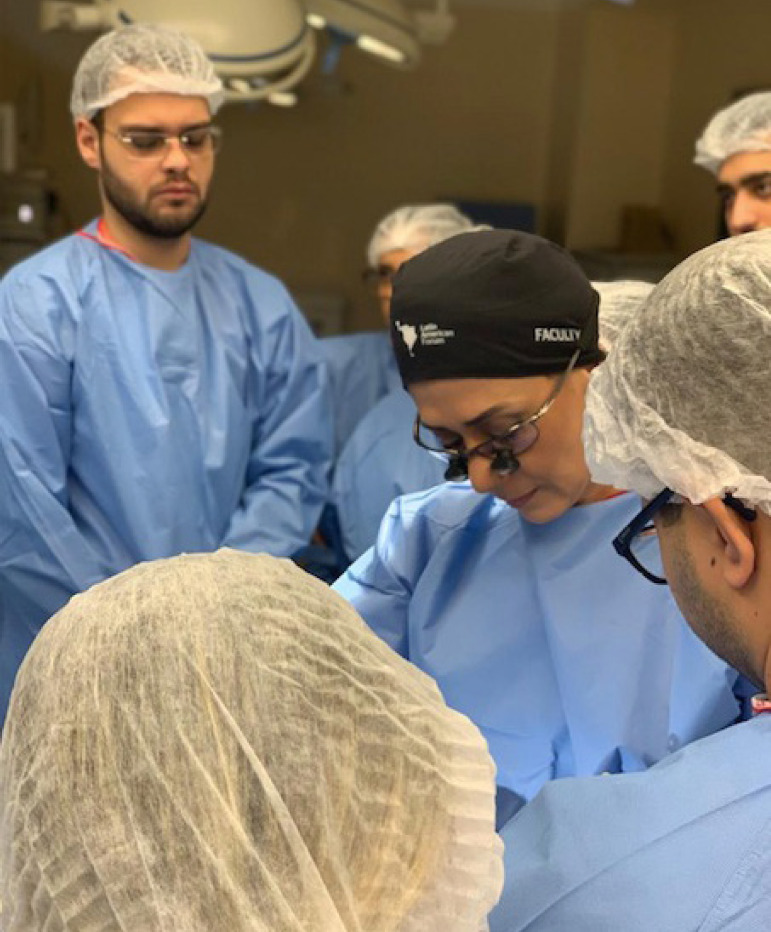
Dr. Magaly Arrais, MD, PhD, in the experimental center teaching surgical techniques to both residents and medical students. Image courtesy from the authors.

Furthermore, annually, the BSCVS organizes the Resident Training in Experimental Surgery (Figure 3) and offers five positions for interested medical students, with the chance of performing and training live surgeries in animal models. The Resident Experimental Surgery’s Training Program was founded in 2010 with the aim to improve skills and surgical technique of cardiovascular surgery’s residents. In 2011, the program was divided in different modules: coronary surgery module, valve module, aortic module, cardiac transplantation module, and minimally invasive surgery module. This program was established and is coordinated by Dr. Magaly Arrais, with 20 open positions for surgical residents associated to the Brazilian Association of Cardiovascular Surgery, five positions for medical students enrolled in the BDALCS, and eight positions for in-training perfusionists, with all the support of BSCVS. The training program happens at the Johnson & Johnson Medical Innovation Institute with the sponsorship of Johnson & Johnson and with the support of Ethicon and Biosurgery, which offers, beyond the surgical training place, surgical instrumentation, suture lines, wires, and video surgery equipment. In 2018, the Training Program officially became part of the BSCVS Academy.

In conclusion, medical school’s curriculum in Brazil does not cover enough about cardiovascular surgery and students must be involved in a lot of extracurricular activities to be broadly exposed to the field. Despite that, education strategies, as already mentioned, as well as the support of BSCVS, are the initial steps for changing the intricate training in cardiovascular surgery.

**Table:** 

Authors' roles & responsibilities	
TSR	Substantial contributions to the conception or design of the work; or the acquisition, analysis, or interpretation of data for the work; drafting the work or revising it critically for important intellectual content; agreement to be accountable for all aspects of the work in ensuring that questions related to the accuracy or integrity of any part of the work are appropriately investigated and resolved; final approval of the version to be published
RMF	Substantial contributions to the conception or design of the work; or the acquisition, analysis, or interpretation of data for the work; drafting the work or revising it critically for important intellectual content; final approval of the version to be published
MAS	Drafting the work or revising it critically for important intellectual content; agreement to be accountable for all aspects of the work in ensuring that questions related to the accuracy or integrity of any part of the work are appropriately investigated and resolved; final approval of the version to be published

## References

[1] Lin J, Reddy RM (2019). Teaching, mentorship, and coaching in surgical education. Thorac Surg Clin.

[2] Preece R, Ben-David E, Rasul S, Yatham S (2018). Are we losing future talent? A national survey of UK medical student interest and perceptions of cardiothoracic surgery. Interact Cardiovasc Thorac Surg.

[3] Rocha RV, Almeida RMS (2018). Cardiac surgery residency in Brazil: how to deal with the challenges of this unique specialty. J Thorac Cardiovasc Surg.

[4] Tekyou Soluções Relação candidato/vaga do concurso de residência médica da Faculdade de Medicina da Universidade de São Paulo.

[5] Scheffer M, Cassenote A, Guilloux AGA, Miotto BA, Mainardi GM (2018). Demografia médica no Brasil 2018.

[6] Fernandes FG, Hortêncio Lde O, Unterpertinger Fdo V, Waisberg DR, Pêgo-Fernandes PM, Jatene FB (2010). Cardiothoracic surgery league from university of São Paulo medical school: twelve years in medical education experience. Rev Bras Cir Cardiovasc.

[7] Tesche LJ, Feins RH, Dedmon MM, Newton KN, Egan TM, Haithcock BE (2010). Simulation experience enhances medical students' interest in cardiothoracic surgery. Ann Thorac Surg.

